# Supplementary material for: Subthalamic beta bursts correlate with dopamine-dependent motor symptoms in 106 Parkinson’s patients
Source: NPJ Parkinsons Dis. 2023 Jan 7;9:2. doi: 10.1038/s41531-022-00443-3 (PMC9825387; doi:10.1038/s41531-022-00443-3)
Supplement: Supplementary file 1 — Supplementary Material [file 41531_2022_443_MOESM1_ESM.pdf]

Supplementary Material

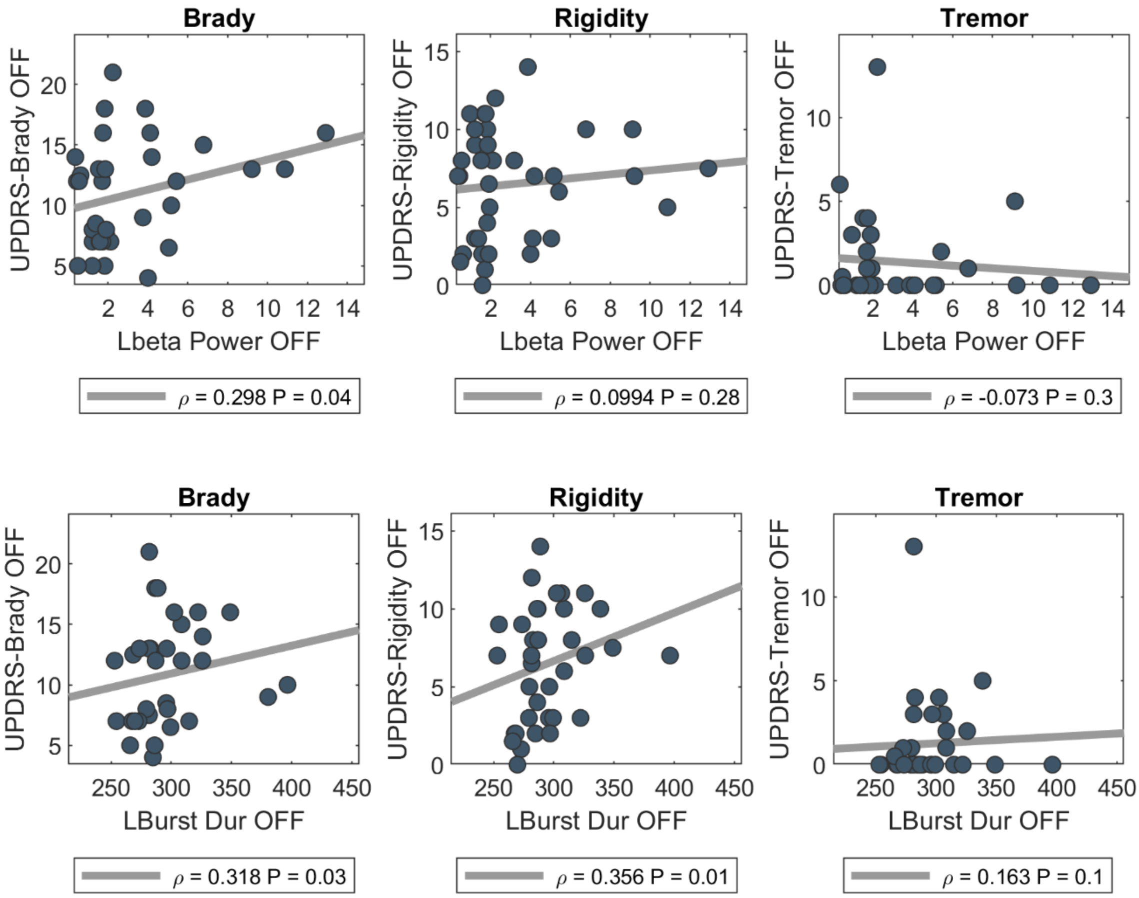

Supplementary Figure 1: LB power and burst duration as symptoms specific marker. Shown are Spearman's correlation coefficients of LB power (upper row) and LB burst duration (lower row) in the OFF medication state with summed scores for the UPDRS-items bradykinesia (item 6.a-9.b, left column), rigidity (item 5, mid column) and tremor (item 3.a-4.b, right column). Not FDR-corrected.

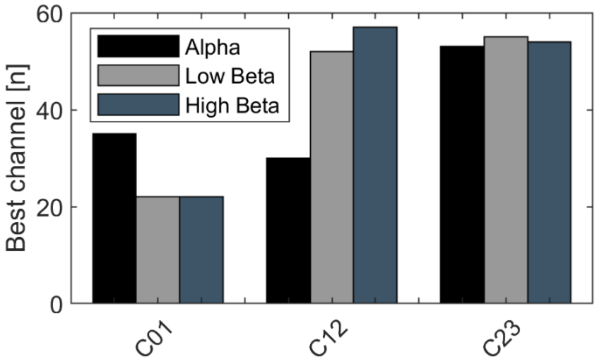

Supplementary Figure 2: Distribution of spectral peaks across contact pairs. Shown is the number of cases in which the most pronounced spectral peak of the respective frequency band (alpha, low beta and high beta) occurred in the lowermost (C01), mid (C21) or uppermost (C23) contact pair.

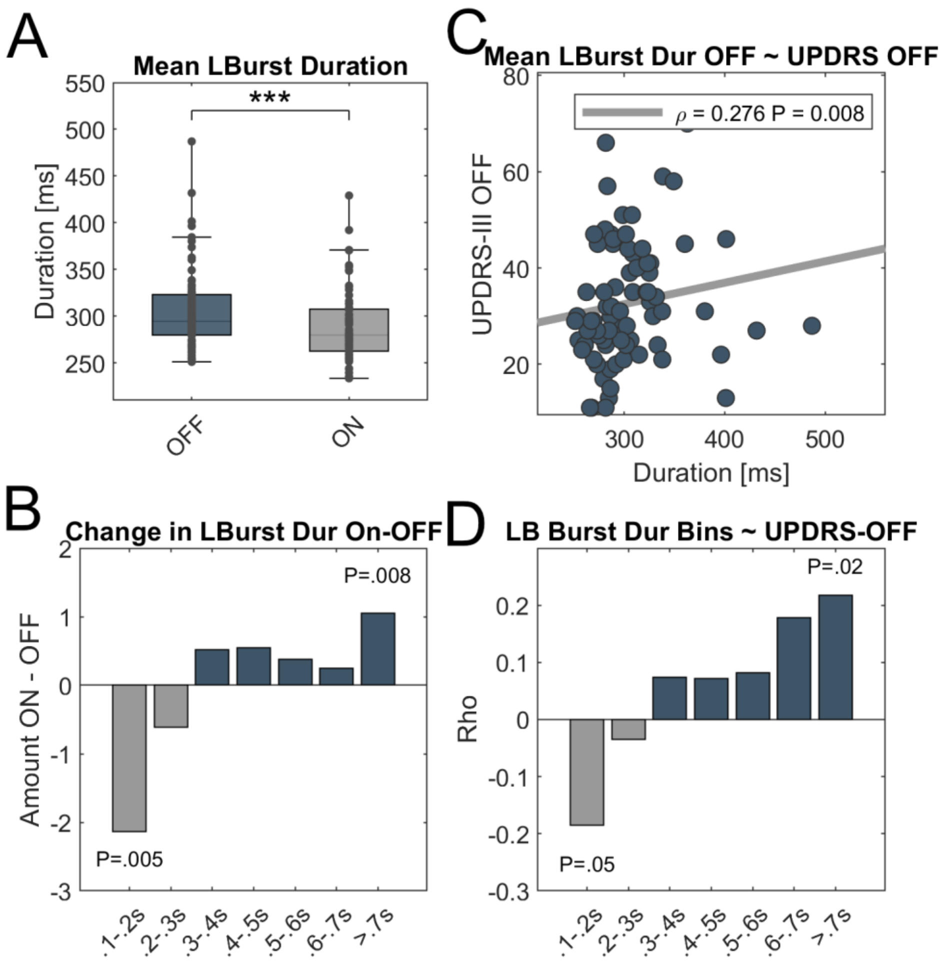

Supplementary Figure 3: Low beta dynamics using the common threshold method based on the 75th percentile of the amplitude distribution. (A) There is a significant shortening of averaged low beta burst duration in the ON-medication state (P<0.001). (B) The increasing burst duration OFF-medication relies on a shift from very short bursts (100-200 ms) to bursts with prolonged duration exceeding 700 ms. Shown is the percentage change from the ON- to the OFF-medication state. (C) Motor impairment shows a significant positive correlation with the averaged LB burst duration. (D) When tested separately, the amount of very long bursts in the OFF-medication state correlates positively with symptom severity (Rho=0.22, P=0.02), while the amount of very short LB bursts show a trend towards a negative correlation with motor impairment. However, both results do not remain significant after FDR-correction. In boxplots, central marks indicate the median and edges the 25th and 75th percentiles of the distribution. *** p<0.001.
