# Supplementary material for: Subthalamic beta bursts correlate with dopamine-dependent motor symptoms in 106 Parkinson’s patients
Source: NPJ Parkinsons Dis. 2023 Jan 7;9:2. doi: 10.1038/s41531-022-00443-3 (PMC9825387; doi:10.1038/s41531-022-00443-3)
Supplement: Supplementary file 2 — Reporting Summary [file 41531_2022_443_MOESM2_ESM.pdf]

nature portfolio

Corresponding author(s): Roxanne Lofredi

Last updated by author(s): Dec 19, 2022

Reporting Summary

Nature Portfolio wishes to improve the reproducibility of the work that we publish. This form provides structure for consistency and transparency in reporting. For further information on Nature Portfolio policies, see our Editorial Policies and the Editorial Policy Checklist.

Statistics

For all statistical analyses, confirm that the following items are present in the figure legend, table legend, main text, or Methods section.

n/a	Confirmed	
☐	☒	The exact sample size (*n*) for each experimental group/condition, given as a discrete number and unit of measurement
☐	☒	A statement on whether measurements were taken from distinct samples or whether the same sample was measured repeatedly
☐	☒	The statistical test(s) used AND whether they are one- or two-sided<br>*Only common tests should be described solely by name; describe more complex techniques in the Methods section.*
☐	☒	A description of all covariates tested
☐	☒	A description of any assumptions or corrections, such as tests of normality and adjustment for multiple comparisons
☐	☒	A full description of the statistical parameters including central tendency (e.g. means) or other basic estimates (e.g. regression coefficient) AND variation (e.g. standard deviation) or associated estimates of uncertainty (e.g. confidence intervals)
☒	☐	For null hypothesis testing, the test statistic (e.g. *F*, *t*, *r*) with confidence intervals, effect sizes, degrees of freedom and *P* value noted<br>*Give P values as exact values whenever suitable.*
☒	☐	For Bayesian analysis, information on the choice of priors and Markov chain Monte Carlo settings
☒	☐	For hierarchical and complex designs, identification of the appropriate level for tests and full reporting of outcomes
☐	☒	Estimates of effect sizes (e.g. Cohen's *d*, Pearson's *r*), indicating how they were calculated

Our web collection on statistics for biologists contains articles on many of the points above.

Software and code

Policy information about availability of computer code

Data collection	Signals were amplified (50.000x) using a D360 amplifier (Digitimer, Hertfordshire, UK) and recorded at a sampling frequency of 1 kHz through a 1401 A-D converter (CED, Cambridge, UK) onto a computer using Spike2 software.
Data analysis	MATLAB code (The Mathworks, Natick, Massachusetts) based on SPM12 (http://www.fil.ion.ucl.ac.uk/spm/) and FieldTrip (http://fieldtrip.fcdonders.nl/).

For manuscripts utilizing custom algorithms or software that are central to the research but not yet described in published literature, software must be made available to editors and reviewers. We strongly encourage code deposition in a community repository (e.g. GitHub). See the Nature Portfolio guidelines for submitting code & software for further information.

Data

Policy information about availability of data

All manuscripts must include a data availability statement. This statement should provide the following information, where applicable:

- Accession codes, unique identifiers, or web links for publicly available datasets
- A description of any restrictions on data availability
- For clinical datasets or third party data, please ensure that the statement adheres to our policy

The data that support the findings of this study are available on request from the corresponding author in the framework of a data sharing agreement. The data are

not publicly available as this would compromise the privacy of research participants according to the current General Data Protection Regulation of the European Union.

Human research participants

Policy information about studies involving human research participants and Sex and Gender in Research.

Reporting on sex and gender	For this study, we included 42 female and 64 male participants. Gender information was based on self report. There was no specific assessment of gender in the reported results except for beta power being similarly pronounced in both genders.
Population characteristics	For this study, we have identified archival local field potential (LFP) data from 106 Parkinson's disease patients (63.5±8 years, 42 female, clinical subtype: 47% akinetic-rigid; 20% tremor-dominant; 33% equivalent) who underwent bilateral implantation of subthalamic deep brain stimulation electrodes.
Recruitment	Parkinson's disease patients undergoing deep brain stimulation surgery at our centers (Berlin, Hannover) were recruited. Parkinson's disease patients that are eligible for deep brain stimulation surgery might have less severe concomittant disease than the overall Parkinson's disease population, which might influence the results. This is further accenuated by the fact that recordings are performed post-operatively. Patients with other medical conditions might be at higher risk to develop post-operative delire and thus less likely to have been included in the study.
Ethics oversight	Ethikkommision der Charité Universitätsmedizin Berlin

Note that full information on the approval of the study protocol must also be provided in the manuscript.

Field-specific reporting

Please select the one below that is the best fit for your research. If you are not sure, read the appropriate sections before making your selection.

☒ Life sciences ☐ Behavioural & social sciences ☐ Ecological, evolutionary & environmental sciences

For a reference copy of the document with all sections, see nature.com/documents/nr-reporting-summary-flat.pdf

Life sciences study design

All studies must disclose on these points even when the disclosure is negative.

Sample size	n=106
Data exclusions	For correlative analyses with UPDRS scores, 11 cases were excluded because UPDRS scores were missing either in the ON or OFF medication state. For analyses of low beta activity, 20 cases were excluded because they did not display a low beta peak. For analyses of high beta activity, 23 cases were excluded because they did not display a high beta peak. For analyses of alpha activity, 24 cases were excluded because they did not display an alpha peak.
Replication	The reproducibility of the results regarding beta burst dynamics was assessed by comparing two different methods for beta burst definition.
Randomization	There was no randomization of the study. The medication state could have been randomized, but this was not possible as it was mainly related to the clinical routine which medication state was recorded first. As we report results from rest activity, randmization to avoid learning effects was not necessary.
Blinding	Given that medication significantly improves motor symptoms in PD, blinding to the condition was not possible for either the patient or the investigator.

Reporting for specific materials, systems and methods

We require information from authors about some types of materials, experimental systems and methods used in many studies. Here, indicate whether each material, system or method listed is relevant to your study. If you are not sure if a list item applies to your research, read the appropriate section before selecting a response.

Materials & experimental systems

n/a	Involved in the study
☒	☐ Antibodies
☒	☐ Eukaryotic cell lines
☒	☐ Palaeontology and archaeology
☒	☐ Animals and other organisms
☒	☐ Clinical data
☒	☐ Dual use research of concern

Methods

n/a	Involved in the study
☒	☐ ChIP-seq
☒	☐ Flow cytometry
☒	☐ MRI-based neuroimaging
